# Development and piloting of an online course to improve knowledge, confidence and attitudes towards triaging images of skin lesions submitted online in primary care

**DOI:** 10.1186/s12909-024-05840-1

**Published:** 2024-08-05

**Authors:** Maria Ntessalen, Albana Krasniqi, Peter Murchie

**Affiliations:** Academic Primary Care Research Group, Institute of Applied Health Sciences, University of Aberdeen, Polwarth Building, AB25 2ZD Aberdeen, Scotland

## Abstract

**Background:**

Melanoma is the 5th commonest cancer in the UK and survivors require frequent and thorough skin checks. During the Achieving Self-directed Integrated Cancer Aftercare (ASICA) trial, melanoma survivors used an app to submit images of concerning lesions for assessment by a dermatology nurse. In the past, online courses have been used to train non-specialist primary care practitioners (PCPs) in this skill.

**Objectives:**

This study aimed to determine whether an online course could increase knowledge, confidence, and attitudes towards skin image triage in PCPs in the Grampian area.

**Methods:**

Preliminary discussions were held with PCPs to determine the need for an online course. The course was designed at the University of Aberdeen and included an introduction to the skin, case studies and quizzes on a variety of skin conditions based on melanoma survivors’ submissions via the ASICA app. Two pre- and post-course questionnaires were administered to all participants to (1) assess knowledge gained and (2) assess any improvements in confidence and attitudes towards triaging skin lesions that could be indicative of skin cancer. All PCPs in the Grampian area were invited to participate with almost 70 medical practices contacted. Results were analysed using a paired sample T-test.

**Results:**

The course was advertised to all GP practices in the Grampian area and 38 PCPs completed all its stages. Undertaking the course improved all PCPs’ confidence and attitudes towards triaging (*p* < 0.001). It also improved knowledge in all non-GP PCPs (*p* = 0.01). Most participants found the course useful; thought it was at the right level of difficulty, right format and thought the design was good.

**Conclusions:**

Our online course in triaging skin lesions submitted digitally to PCPs was able to improve knowledge, confidence, and attitudes towards triaging. The course was acceptable in its design and was deemed useful and applicable to practice. Further research should investigate the effect the course has on secondary care referral numbers.

## Significance

**What is already known about this topic?:**

Skin problems can make up to 15% of Primary Care appointments and patients with benign or low risk skin problems are often inappropriately referred to specialists causing delay in secondary care.

Based on the findings of the ASICA trial it was suggested that low risk concerns can be effectively triaged by non-specialist primary care practitioners (PCPs).

Previous studies showed that online courses can improve knowledge, confidence, and attitudes towards triaging images of skin lesions.

**What does this study add?:**

The content of this online course was designed based on the most commonly submitted lesions in the ASICA trial and allowed PCPs (including GPs, GP trainees, ANPs, practice nurses and physician associates) to complete it in their own time.

The study showed that completing the online course increased PCP confidence and attitudes towards triaging skin lesions, regardless of their prior knowledge and experience.

The course also increased knowledge of all non-GP PCPs as well as PCPs with up to 2 years in their current post.

## Introduction

Melanoma is a cancer of pigment-producing cells within the skin and can result from long-term exposure to UV light [1]. It is the fifth commonest cancer in the UK and incidence is increasing, making it a significant public health concern [2, 3]. 10 year recurrence rates for patients treated are reportedly high beginning from 20% for stage IB/ IIA melanomas (accounting for almost 28% of all primary melanomas) and up to 67% for stage IIB/C primary melanomas (accounting for almost 10% of melanomas) [4]. These patients require regular monitoring to diagnose melanoma at an early stage as early diagnosis offers better treatment outcomes [4, 5]. However, the need for regular monitoring through skin checks in secondary care may be disadvantaging certain groups, such as rural dwellers, as it may mean longer journey times to the hospital and therefore could result in poorer outcomes [6, 7]. Additionally, given the shortfall of dermatologists in the UK and increasing skin cancer workload, it is imperative primary care practitioners (PCPs) work together with secondary care to help meet increasing demands [7, 8].

In recent years, digital healthcare has been increasingly used as a method for skin lesion triaging to limit unnecessary hospital attendance and to help patients facing difficulties accessing care due to geographic location [9, 10]. In line with this, the Achieving Self-directed Integrated Cancer Aftercare (ASICA) app was developed to help melanoma patients with their monthly skin checks to aid early detection of recurrent or new melanoma [11, 12]. The app prompted and supported Total Skin Self Examinations (TSSEs) and provided participants with the opportunity to contact a Dermatology Nurse Practitioner (DNP) and receive feedback on any worrisome skin problems they had [13]. Participants in the ASICA trial were randomised to the ASICA intervention plus standard care, or standard care alone in a 1:1 ratio using a validated remote computer-automated randomisation system hosted at the Centre for Healthcare Randomized Trials (CHaRT) in Aberdeen [11, 12]. 120 participants used ASICA for 12 months and together submitted a total of 189 concerns with their skin to be checked by the trial dermatology specialist nurse during that period. Participants were given a tablet with a built-in digital camera preloaded with the ASICA app and received comprehensive training on how to use the app (in person, group and written instruction). No restrictions were made on the nature of skin concerns that they should report as patients all had prior experience of receiving melanoma follow-up examinations [11, 12]. Most concerns submitted were low risk and could be resolved without the need to see the participant face to face. The DNP could usually resolve the concern by referring to images and text descriptions submitted by the patient and following up with them by telephone, sometime requesting follow-up images. A relatively small number of patients, with higher risk concerns, required to be seen face to face; 7% triggered face-to-face consultations with a General Practitioner (GP) and 10% were seen at a dermatology clinic.

During analysis of qualitative data from the DNP in the ASICA trial (unpublished data), it was suggested that many low risk concerns could be effectively and efficiently triaged by non-specialist PCPs. This could have the added advantage of offering quicker reassurance for patients and enable more efficient use of specialist time to focus on higher risk concerns. The potential is further emphasised by the fact that initial screening of skin problems is a core activity for PCPs, where approximately 15% of primary care appointments are related to the skin [14]. Not surprisingly a previous systematic review has suggested that the diagnostic accuracy of pigmented lesions is lower for primary care physicians than dermatologists [0.42-1.00 vs. 0.81-1.00] so it is important that further effective training in skin-lesion triage is made available to PCPs to support them in this role [3].

A systematic review found that brief online courses can increase confidence and knowledge of PCPs skin lesion triage [15, 16]. Currently, however, there are few well-designed and evidence-based courses that support the development of skin problem triage skills by PCPs [17, 18]. To address this we designed an online course informed by the concerns submitted by participants in the ASICA trial. Our aim was to determine whether this course could increase the knowledge, confidence and positive attitudes toward skin lesion triage of relevant non-dermatology specialist PCPs such that they could have a larger role in the widescale implementation of ASICA into National Health Service (NHS) practice.

## Methods

### Development of the course

One-to-one preliminary discussions were held with 3 Advanced Nurse Practitioners (ANPs) working in primary care in the NHS Grampian area in January 2022 to scope how useful they would perceive a course in skin lesion triage to be. Discussions focused on: (1) The current situation in Primary Care, (2) How well-equipped PCPs felt to carry out triaging, (3) How they triaged the lesions that would come to them, (4) What would be the best way to train PCPs. The discussion took place online.

### Course content

The content of the course was informed by the results of the ASICA trial with the commonest diagnoses forming its basis [12]. The course consisted of 2 main sections. The first provided a general introduction to the skin, discussed skin cancer, skin protection and assessment of suspicious skin lesions using the ABCDE algorithm - a mnemonic developed to help patients and physicians identify possible melanomas early where A is for Asymmetry, B is for Border irregularity, C is for Colour variability and/or Changing colour, D is for Different, E is for Evolving (changing) [19]. This was chosen above the modified Glasgow Algorithm as despite both being validated tools for assessing pigmented skin lesions (), as the former tool was deemed to be more memorable and easier to use given it is already in a checklist/ mnemonic format.

Each case study consisted of a patient scenario intentionally presented in an e-consult format to provide a realistic reflection of how skin lesion triage scenarios may present in primary care day-to-day. E-consult cases were formatted in a way that guided participants through clinical assessment of the lesion, assessment of level of concern, management steps and the appropriate outcome. Eleven scenarios were included in the course with primary diagnoses being (1) cherry haemangioma, (2) squamous cell carcinoma (SCC), (3) basal cell carcinoma (BCC), (4) dermatofibroma, 5)skin tag, 6) seborrhoeic keratosis, 7) melanoma, 8) atypical melanocytic naevus, 9) benign melanocytic nevus, 10) benign melanocytic nevus and 11) subungual haematoma. The case studies were interactive and required participants to provide answers before receiving feedback. Cases were grouped into sections and after a few cases, participants were asked to answer MCQs relating to the cases in the section they had just completed. Each quiz question depicted four images of lesions, three correct and one differential (incorrect) answer, and feedback using ABCDE descriptors was provided upon answer selection. There was a total of 11 case studies and 27 MCQs.

Course content was written up by the authors of this paper. Course content and questionnaires were reviewed for validity of content and proofread by three GP colleagues and a final year medical student with special interests/ experience in dermatology, two research fellows and two research assistants. Feedback on the correctness of content, structure and language was provided. Unfortunately, no Dermatology Consultants in the area were available provide feedback on this.

Moodle, an online platform used by Aberdeen University Medical School, was used to host the course. Material was transferred to Moodle by a software engineer at Aberdeen University. The course was designed to take approximately 3–4 h to complete however PCPs were allowed to complete it over 6 weeks.

As is standard on Moodle courses by the medical school, all participants were asked to complete an evaluation form at the end of the course to provide feedback on the dermatology case studies.

### Recruitment and participants

A member of the NHS Research Network (NRS) Primary Care Network contacted all practice managers in the Grampian area with information about the online course (January 2023). Practice managers were asked to circulate an email to PCPs. Within this study PCPs included GPs, GP trainees, ANPs, practice nurses and physician associates. A reminder was circulated 2 weeks later to notify PCPs of the final date and prompt them to join the course if they were interested. Personal networks were also contacted.

### Course evaluation methods

Participants emailed the course coordinator (MN) expressing their interest and were subsequently sent two pre-course questionnaires to complete which measured knowledge, confidence and attitudes towards triaging skin lesions. A 12-item questionnaire was developed by one of the authors (AK), assessing knowledge in triaging skin lesions using MCQs. No feedback was given to participants regarding their answers. A second 33-item questionnaire was developed by two of the authors (AK, PM), to collect personal and professional information and to assess confidence and attitudes towards triaging skin lesions on a scale from 1 to 10, with 1 being the least confident and 10 being the most. Following completion of the questionnaires, access was given to the course and participants were asked to complete it within 6 weeks. REDCap 13.1.25, an online database with in-built ability to create surveys, was used to design and distribute the pre- and post-course questionnaires.

Participants who did not complete the questionnaires within 2 weeks were sent two reminders 1 week apart as were those who had not started it within 2 weeks. All participants received a reminder email at 4 weeks since they were given access to the course and again a week later.

Following completion of the course, participants notified the course organiser and received the final two questionnaires. One was identical to the pre-course questionnaire. The other assessed confidence levels and attitudes towards triaging skin lesions.

The questionnaire was identical to the one they received at the beginning of the course, minus questions on personal information. Questions on confidence were assessed using a sliding bar. The participant chose how they felt about the question asked (using the sliding bar) and that automatically gave a number from 1 (least confident) – 10 (most confident). Confidence question examples included “How confident are you that if you find an issue of concern on an image of a skin lesion that you will take appropriate action?”. Questions on participants attitudes towards triaging skin lesions were scored using a 5-point Likert scale varying from strongly disagree- disagree- unsure- agree- strongly agree. Strongly disagree was given a value of 1 and strongly agree was given a 5. Attitude question examples include “It is important to carefully assess skin lesions submitted by patients?” and “I could find suspicious features on an image of a skin lesion if they were there.”

The values were added up before and after the course was taken to estimate a confidence score and a score on attitudes.

### Statistical analysis

The pre- and post-course questionnaires were designed and sent using REDCap. After receiving all responses, the data were exported in cvs format and analysed using SPSS 28. The data were assessed for normal distribution and the appropriate test was applied (in this case a paired sample t-test). A p value of 0.05 was set as the point of significance. Only data from participants who completed both the pre- and post- test questionnaires were included in analysis.

## Results

### Participants

**Fig. 1:**
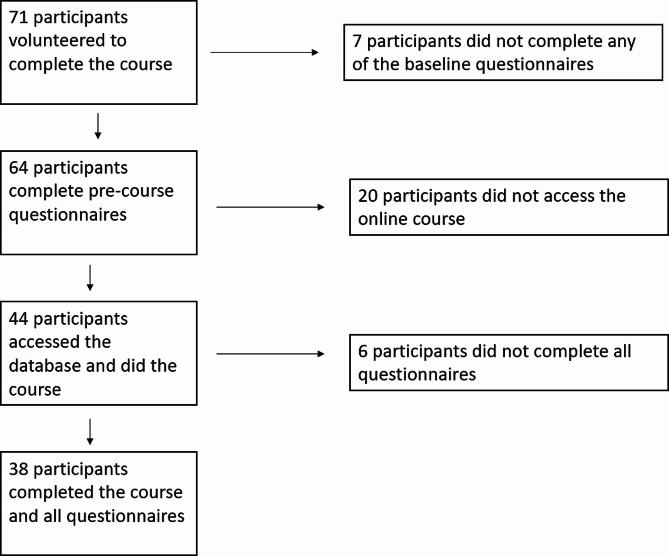
Flow diagram showing the number of people who joined the course and the number of people who were able to complete it and submit all questionnaires

All GP practices in Grampian, Northeast Scotland, were invited. Seventy-one PCPs contacted the course coordinator and expressed their interest. Figure 1 shows the number of people who joined the course and completed all steps. Thirty-eight out of 71 participants (53%) completed the course and all steps involved.

Table 1. shows characteristics of the participants who completed the online course. The participants were asked a number of questions at baseline that pertained to their age, gender, job role, years in their current post and previous training in dermatology and triaging.

**Table 1:** Participant characteristics and training in dermatology and triaging

Parameters measured	Results *n* = 38
Age range (years) (% of total)	• 18–30 (5.3%) • 31–45 (57.9%) • 46–54 (18.4%) • 55–65 (18.4%)
Gender n (% of total)	• 29 (76.3%) female • 8 (21.1%) male • 1 (2.6%) did not answer
Job role (n, %)	• Primary care nurse (3, 7.9%) • ANP daytime (11, 28.9%)* • ANP out of hours (1, 2.6%) • GP (23, 60.5%) • Other (1, 2.6%) (PA^3^) * One nurse occupied two posts ANP daytime and ANP OOH
Duration in current post, range (n, %)	• 0–2 (8, 21.1%) • 2–5 (13, 34.2%) • 5–10 (7, 18.4%) • 10–15 (1, 2.6%) • 15 or more (9, 23.7%)
Have you had formal training in dermatology? (n, %)	• Yes (12, 31.6%) (no ANPs had training in dermatology) • No (26, 68.4%)
Types of training in Dermatology (n)	• SHO (3) • GPST rotation (5) • Undergraduate medical school (5)
Have you had specific training to undertake triaging (of skin lesions)? (n,%)	• Yes (5, 13.2) (2 ANPs had training in triaging) • No (33, 86.8%)

SHO, Senior House Officer^1^; GPST, General Practice Specialty Trainee; ANP, Advanced Nurse Practitioner^2^; PA, Physician Associate^3^

^1^ A hospital doctor, typically on their 1st or 2nd year after graduating medical school

^2^ A nurse with Level 7 MSc qualification in clinical assessment and a prescribing qualification which enables them to make autonomous decisions in the assessment, diagnosis and treatment of patients

^3^ Physician associates are generalist healthcare professionals who have both a relevant degree or masters degree plus 2 year postgraduate diploma

Participants were also asked about frequency of triaging lesions, number of lesions triage and areas most commonly assessed (Table 2).

**Table 2:** Frequency and number of lesions triaged and body areas most assessed

Do you triage skin lesions? (*n*, %)	• Yes (28, 73.7%) • No (9, 23.7%) • Did not answer (1, 2.6%)
How often do you triage skin lesions? (n, %)	• Daily (9, 23.7%) • Weekly (15, 39.5%) • Less than monthly (1, 2.6%) • Monthly (2, 5.3%) • Other (1,2.6%) • Did not answer (10, 26.3%)
How many skin lesions do you triage in a month? (n, %)	• 0–2 (2, 5.3%) • 2–5 (16, 42.1%) • 5–10 (6, 15.8%) • 10 or more (4, 10.5%) • Did not answer (10, 26.3%)
How often do you triage skin lesions and make decisions about them? (n,%)	• Never (1, 2.6%) • Rarely (1, 2.6%) • Monthly (4, 10.5%) • A couple of times a month (10, 26.3%) • Weekly (20, 52.6%) • Daily (2, 5.3%)
In the next 12 months, how many times do you think you will assess skin lesions and make decisions about it? (n,%)	• Rarely (1, 2.6%) • Monthly (3, 7.9%) • A couple of times a month (14, 36.8%) • Weekly (19, 50%) • Daily (1,2.6%)
Thinking back to the last time you assessed a skin image submitted to the practice by a patient, which areas of the body did you assess? (n)	• Face (27) • Neck (12) • Upper chest (13) • Arms (17) • Hands (5) • Torso (14) • Front of thighs/ knees/ shins (7) • Feet (5) • Back of thighs/ knees / shins (5) • Bottom (4) • Lower back (8) • Upper back (10) • Back of neck/ scalp (9)

### Course evaluation

Participants completed two questionnaires during course evaluation. A questionnaire assessing knowledge before and after they took the course, and a questionnaire assessing confidence and attitudes towards triaging skin lesions at the same time points. Table 3 shows mean values (SD) of questionnaires for the whole cohort and for different sub-groups. Notably, confidence increased significantly for the whole cohort, whereas knowledge increased significantly for non-GP PCPs and participants with just 0–2 years in their current post.

**Table 3:** Pre- and post-course scores ± SD in knowledge and confidence in the whole cohort and in sub-groups

	Pre-course score	Post-course score	*P* value
**Questionnaire assessing knowledge**			
Knowledge score, *n* = 38	7.38 ± 2.08	8.18 ± 1.96	0.056
Knowledge score, *n* = 21 (GPs only)	8.05 ± 1.96	8.38 ± 2.17	0.611
Knowledge score, *n* = 19 (all PCPs apart from GPs)	6.63 ± 2.00	7.95 ± 1.71	**0.010**
Knowledge score in participants with 0–2 years in their current post, *n* = 7	6.43 ± 1.90	8.57 ± 1.52	**0.008**
Knowledge score in participants with 2–5 years in their current post, *n* = 12	7.67 ± 1.92	8.67 ± 1.82	0.172
Knowledge score in participants with 5–10 years in their current post, *n* = 7	7.57 ± 1.90	6.43 ± 1.61	0.268
Knowledge score in participants with 15 + years in their current post, *n* = 9	8.00 ± 2.34	8.00 ± 2.17	1.000
**Questionnaire assessing confidence and attitudes towards triaging**			
Confidence and attitudes score, *n* = 34	58.19 ± 6.75	68.57 ± 7.10	**< 0.001**
Confidence score GPs only, *n* = 20	25.65 ± 4.63	32.30 ± 4.61	**< 0.001**
Confidence score all PCPs apart from GPs, *n* = 14	25.43 ± 4.30	32.79 ± 4.80	**< 0.001**
Attitudes score GPs only, *n* = 20	33.35 ± 2.75	36.10 ± 3.09	**< 0.001**
Attitudes score all PCPs apart from GPs, *n* = 9	31.56 ± 5.05	37.22 ± 2.68	**0.017**

Participants feedback on course difficulty, usefulness, and overall satisfaction are seen in Table 4. Feedback from all participants who started the course and attempted at least one question was analysed as feedback from non-completers was felt to be helpful in giving insight into barriers to course completion/ negative aspects to course design which could be useful for future improvement of the course/ similar courses.

Further information collected from the baseline questionnaire on confidence and attitude towards triaging skin lesions includes comments left by PCPs in an open textbox. Before joining the course, participants provided comments such as the following:

“Worried that I mainly rely on my experience of seeing abnormal lesions in past to guide my decision of whether lesion is abnormal rather than any “guideline” based decision making “ (Female, 46–54, GP).

The time pressures in General Practice are so great at the moment that what I would want to do in an ideal world is not necessarily what I have time for currently. It would be good to look at every lesion in person and take a full history but if a patient has submitted a photo of a suspicious looking lesion I am more likely simply to refer on to dermatology. (Female, 55–65, GP)

According to participant feedback post-course, it was “useful”, “educational” and allowed them to “gain knowledge which they took directly back to practice”. Participants also found use of cases and MCQs very useful for learning and liked the realistic set-up of the course with the presentation of the e-consult.

**Table 4:** Feedback from participants who started the course and attempted at least one question

Question	Answers and percentages
How difficult was the case? (*n* = 45)	• Too difficult (0%) • About right (100%) • Too easy (0%)
How useful was the course? (*n* = 46)	• Useful (86.96%) • Of some use (13.04%) • Not useful (0%)
Do you think case-based learning is generally useful? (*n* = 44)	• Yes (100%) • No (0%) • Don’t know (0%)
Would you like to see more case-based learning? (*n* = 43)	• Yes (95.35%) • No (2.33%) • Don’t know (2.33%)
Overall, I found the design (*n* = 46)	• Good (76.09%) • Average (21.74%) • Poor (2.17%)

Participants also provided opinions on the course in a free-text question (Table 5). These focused on suggestions to improve technical issues and praise for the course. The technical feedback was on an error that appeared during the release of the course where images on a case were no longer available. A small number of participants fed back that images could be larger to aid with identification of diseases. One participant felt not all topics in MCQs were covered in case studies. Another suggested a navigation bar plus the ability to go back to where they had left off would be a good addition to the course. A few participants suggested increasing the number of MCQs and providing more feedback on the “incorrect” answers to questions. Finally, more than a third of participants expressed how useful they found the course in increasing their knowledge, how it would help their practice and that they would like to see more of it.

**Table 5:** Summary of positive and negative feedback from free-text question

Positive	Negative
Very useful course	Technical issues - ensure images always appear in case studies, increase image size to help with identification, include a navigation bar and the ability to continue from where course stopped
Helped improve their practice	Increase the number of MCQs and provide more feedback on the differential diagnosis
Appetite for more courses on examining skin lesions	Include all topics from MCQs in case studies

## Discussion

### Summary of findings

This study developed and evaluated a digital healthcare intervention in the form of an online course which aimed to improve the knowledge, confidence and attitudes of PCPs in triaging digital images and descriptions of skin concerns submitted by patients to support self-directed total-skin-self-examination. Of the PCPs who volunteered to take the course and complete the pre- and post-course questionnaires, 38 submitted all questionnaires and completed the course (53%). The training provided during the course increased confidence and attitudes in the cohort of PCPs (*p* < 0.001). Knowledge increased in the whole cohort but was only significantly increased when GPs were excluded from the analysis (*p* = 0.010) or among PCPs with only 0–2 years of experience in their current post (*p* = 0.008).

### Context with other research

A number of online courses were previously designed aiming to improve the dermatological skills and practice of PCPs as already mentioned. Their content is variable, either covering only specific areas of dermatology/ body sites [20, 21] or being based on referral guidelines determined by “red flags” [22]. The ASICA trial specifically analysed cases that were frequently encountered by a dermatology nurse practitioner as reported by participants. Consequently, in addition to covering cases centred around a single topic, the course provided comprehensive information and training on skin cancers and pigmented lesions, which has been proven to enhance the diagnostic accuracy of PCPs [23] .

In our study, we gathered information about training received by PCPs prior to attending the course. It was notable that out of the 38 PCPs, 26 (68.4%) had no prior training in dermatology. Specifically, none of the non-GPs had received any training (11 /11), and more than half of GPs had also received no training (15/27). Interestingly, despite the lack of formal training, a significant proportion of PCPs reported regularly triaging images submitted to their practice. Over 40% of PCPs reported triaging images on a weekly basis, 15% reported triaging 5–10 images per month and more than 10% reported triaging 10 or more images per month.

Our study assessed participants’ knowledge in dermatology before and after the course but despite an increase in mean knowledge (before: 7.38 ± 2.08 vs. after: 8.18 ± 1.96) the difference was not statistically significant (*p* = 0.056) for the whole cohort. Assessment of pre and post course knowledge was via completion of 12 MCQs on skin anatomy, risk factors for skin cancers and differential diagnoses. Similarly, other questionnaires which have been used to test PCP knowledge on skin conditions also included between nine [24] and thirty questions [25]. Once GPs were excluded from the analysis, other PCPs did demonstrate a significant increase in knowledge suggesting those with no previous formal dermatological training have much to gain from our course. This course may be more effective overall for non-GP PCPs (ANPs/ primary care nurses/ PAs) than GPs as it has achieved a significant increase in their knowledge level despite number of years in their current role. However, GPs did appear to report increased confidence after undertaking the course.

The course increased confidence in everyone and knowledge in the non-GP cohort showing that allowing the PCPs to take the course at their own pace was still beneficial to them. A number of other courses have been carried out on different time scales varying from 1 h [26] to 24 months [27]. Our course shows that increases in knowledge and confidence can still be achieved even if PCPs personalise their engagement with the material to their own schedules. This approach reflected engagement with the course in a pragmatic manner as daily pressures can make daily or even weekly engagement difficult. An important parameter that was not explored in our study was the maintenance of knowledge and confidence long-term. In other studies where knowledge and confidence were assessed long-term the average value remained increased at 6 months but dropped by 12 months [28, 29], perhaps suggesting the need for refresher courses. Supporting this, an RCT that evaluated whether a short dermoscopy e-learning course (4 h) was non-inferior to a longer course (12 h) in terms of PCPs’ competence in selective triage of skin tumours found spaced test-based refresher training sessions appear to maintain and even increase the skills acquired by PCPs over time [30].

While only 53% of participants completed the course, this compares favourably with completion rates for other similar online courses aimed at primary care professionals for whom competing demands and workloads are high. For example, a French study by Greco et al. (2023) aiming to evaluate knowledge on the diagnosis and management of common nail conditions using a 31-minute online training session was deemed successful with an identical 53% completion rate. Furthermore, of the 47% of participants who did not complete our course, only 15% actually accessed the course at all suggesting that barriers to completing the course were unlikely to be due to intrinsic limitations of the course. The remainder who started but did not complete the course may have done so for various reasons including workload pressures.

### Strengths and weaknesses

Our study is the first to create an online course in triaging skin lesions submitted to PCPs informed directly by their needs and supported by the most commonly found skin lesions as observed in the ASICA trial. This course consisted of an interactive set-up with case-studies and MCQs which proved to be well received by participants and was useful in their learning and understanding. We demonstrated the course was attractive to busy PCPs, and that they could practically complete and benefit from it within a timescale which would be realistic for widescale implementation within the NHS.

As our study depended on volunteers, the PCPs who enrolled may not have been the most representative of PCPs tasked with skin lesion image triage in general. A number of PCPs mentioned a personal interest in dermatology and attendance of every opportunity for training on the field. As our cohort included all PCPs in Primary care, we were unable to have a representative sample from all divisions (GPs, ANPs, practice nurses etc.) and some were under-represented introducing bias (60% of participants were GPs) and limiting sub-group analysis.

### Future research

Our course has demonstrated short-term potential to increase the knowledge and confidence of those who are increasingly being tasked with the triage of skin lesion images in primary care. Future research should look to define the best way to consolidate and sustain these improvements. Additionally, we did not look at the impact the course had on clinical practice. Future studies could also look into specificity and sensitivity of triaging by PCPs and the number of referrals to secondary care before and after training.

## Conclusion

This online course was able to increase knowledge and confidence in the PCPs who undertook it and appeared to be acceptable in its design, level of difficulty and usefulness and could be completed within a time commitment practical for busy PCPs. The course appeared to be particularly effective in improving scores for those with less than two-year’s experience, suggesting it may have value for those PCPs in training, and/ or new to skin lesion triage. While the content of this course was initially based on data from submissions to the ASICA trial which aimed to support detecting melanoma early in survivors, it included cases on benign lesions and non-melanoma skin cancers as differentials and in order to equip participants to triage a broad range of skin lesions/ concerns. Therefore, the knowledge gained from our course can be used in the triage of skin concerns in any patient in primary care where PCPs are increasingly being tasked with the triage of skin lesion images. Further research should look to characterise the effect this course has on the number and nature of referrals to secondary care.

## Data Availability

Data can be made available following request to Professor Peter Murchie (p.murchie@abdn.ac.uk).
